# Brain-machine interfaces can accelerate clarification of the principal mysteries and real plasticity of the brain

**DOI:** 10.3389/fnsys.2014.00104

**Published:** 2014-05-26

**Authors:** Yoshio Sakurai

**Affiliations:** Department of Psychology, Graduate School of Letters, Kyoto UniversityKyoto, Japan

**Keywords:** brain-machine interface, neuronal coding, cell assembly, functional localization, ongoing activity, brain-body interaction, brain plasticity

## Abstract

This perspective emphasizes that the brain-machine interface (BMI) research has the potential to clarify major mysteries of the brain and that such clarification of the mysteries by neuroscience is needed to develop BMIs. I enumerate five principal mysteries. The first is “how is information encoded in the brain?” This is the fundamental question for understanding what our minds are and is related to the verification of Hebb’s cell assembly theory. The second is “how is information distributed in the brain?” This is also a reconsideration of the functional localization of the brain. The third is “what is the function of the ongoing activity of the brain?” This is the problem of how the brain is active during no-task periods and what meaning such spontaneous activity has. The fourth is “how does the bodily behavior affect the brain function?” This is the problem of brain-body interaction, and obtaining a new “body” by a BMI leads to a possibility of changes in the owner’s brain. The last is “to what extent can the brain induce plasticity?” Most BMIs require changes in the brain’s neuronal activity to realize higher performance, and the neuronal operant conditioning inherent in the BMIs further enhances changes in the activity.

## Introduction

A brain-machine interface (BMI) is used to enable the neuroprosthetic control of external devices by neuronal activity instead of body parts movements (Lebedev and Nicolelis, 2006; Berger et al., 2008; Hatsopoulos and Donoghue, 2009; Nicolelis and Lebedev, 2009; Andersen et al., 2010; Moran, 2010; Green and Kalaska, 2011; Lebedev, 2014). Although the development of invasive BMIs has been making a steady progress and holds promises for future clinical use (Lebedev and Nicolelis, 2011; Lebedev et al., 2011; Nicolelis, 2011; Ethier et al., 2012; Hochberg et al., 2012; Collinger et al., 2013), currently available BMIs are limited in terms of accuracy and efficiency with which they can be controlled. As described in the papers referenced above, it is possible to indicate some technical factors affecting the limited performance of current BMIs. However, as also emphasized in some of the papers (e.g., Nicolelis and Lebedev, 2009; Andersen et al., 2010), improvements in the technical factors alone cannot solve all the problems preventing the realization of an ideal BMI, i.e., a system controlling external neuroprosthetic devices freely as intended by the brain without any special training. The ideal BMI required rich and precise information that depends on the activity and function of the brain. Therefore, as Nicolelis (2003), Baranauskas (2014), and Mandonnet and Duffau (2014) has discussed, knowledge of what the brain is and how it works, the ultimate goals of neuroscience research, are essential for BMI research. To achieve these goals, the present paper enumerates five principal mysteries of the brain that must be clarified. It should be emphasized that BMI research has the potential to clarify these principal mysteries and, at the same time, their clarification by neuroscience research is necessary to realize the ideal BMI.

## How is information encoded in the brain?

As the final goal of a BMI is to detect neuronal activity representing information in the brain, BMI research inevitably faces the problem of how is information encoded in the working brain. Neuronal coding (e.g., Calvin, 1996; Abbott and Sejnowski, 1999; Nicolelis, 2001; Nicolelis and Ribeiro, 2006; Holscher and Munk, 2009) is one of the principal mysteries of the brain and may be the ultimate problem of neuroscience, because its final goal is to bridge the mind and brain and detect the mind from brain activity. The early studies of BMIs (Chapin et al., 1999; Wessberg et al., 2000; Nicolelis and Chapin, 2002) have already produced very important and instructive findings demonstrating the nature of the neuronal coding of information. They reported that the activity of only a limited number of neurons randomly sampled from the motor cortex of an animal provided sufficient information to predict arm kinematics during reaching, as well as hand gripping force. In addition, the accuracy of prediction increased as the number of recorded randomly sampled neurons increased. These results indicate that kinematic and kinetic parameters are coded not by the activities of specific motor-related neurons but by the activity of many neurons distributed in the motor cortex. Subsequent BMI studies more or less supported this notion of neuronal coding in the motor cortex (e.g., Carmena et al., 2003). Therefore, as Nicolelis (2003) and Nicolelis and Lebedev (2009) have suggested, a BMI both utilizes population coding by cell assemblies (Hebb, 1949), functionally connected neurons acting as codes representing information in the working brain (Eichenbaum, 1993; Sakurai, 1996b, 1999; Harris, 2005; Sakurai and Takahashi, 2006, 2008; Buzsáki, 2010; Wallace and Kerr, 2010; Sakurai et al., 2013), and provides new insights on this coding. In other words, the theory of cell assembly has been further verified by BMI studies and is approaching an answer to the mystery of neuronal coding.

Although recent neuroscience studies have often reported small populations of neurons related to information processing (e.g., Takahashi and Sakurai, 2009a,b; Opris et al., 2012, 2013) and BMI research has clearly supported the cell assembly theory, the existence of cell assemblies as carriers of neuronal codes has not yet been directly proven, because current BMIs have a bias in the firing rate or amplitude of neuronal activity used as the source signals. This bias may be a factor affecting the limited performance of current BMIs (Sakurai et al., 2014). According to the notion of cell assembly, synchronous and oscillatory activities among many neurons may have the potential to be informative signals for BMIs. It is expected to construct a BMI system which uses ensemble and correlated firing of distributed many neurons, in addition to their firing rates, as neuronal source signals.

## How is information distributed in the brain?

BMI studies have revealed the fact that the neurons whose activity can be used as signals representing information of motor movements are distributed in the motor cortex. Concerning the range of distribution of such neurons, some BMI studies have obtained an optimal basis for brain control of devices by recording the activity of neurons in the precentral (motor) cortical area associated with actual limb movement (Chapin et al., 1999; Taylor et al., 2002; Carmena et al., 2003; Hochberg et al., 2006; Koike et al., 2006; Choi et al., 2009). However, some other studies on BMIs demonstrated their ability to predict movements from neurons in the postcentral (parietal) as well as the precentral cortical areas (Wessberg et al., 2000; Carmena et al., 2003). Although precentral motor neurons can provide accurate predictions of force and displacement even in small numbers (Koike et al., 2006; Choi et al., 2009), many neurons from the parietal and other cortical areas could also have the potential to provide significant predictions. The prediction accuracy increased with the number of neurons included, even when the included neurons were randomly selected from the non-motor area and unrelated to motor movement in nature (Wessberg et al., 2000; Carmena et al., 2003). This indicates that neuronal information on motor movements and forces is widely distributed in cortical areas.

These findings by BMI studies could challenge the classical and conservative view of functional localization based on functional divisions in the brain. Constructing functional divisions is a major problem of neuroscience and many researchers are investigating what functions are localized in what brain areas. The results of BMI studies indicate that the functional boundaries are not definite and fixed but obscure and dynamic. Some BMIs do not necessarily require the selection of functionally specific motor neurons (e.g., Moritz et al., 2008) or, as described above, a specific motor area in the brain to improve their performance in brain control of devices. This notion may be related to the theory of multipotentiality of the brain (John, 1980). This theory suggests that any neuron and region may contribute to the mediation of a diversity of functions and that many neurons and regions contribute to many functions, although it does not imply that different neurons and regions have complete equivalence of functions or that different functions depend equally on diverse neurons and regions. BMI research may again direct the spotlight on the theory of multipotentiality and push back the view of too rigid and too subdivided functional maps. On the other hand, regarding the use of a BMI as a neuroprosthetic system, it is advantageous for it to have the potential to utilize any neuron and any brain region unrelated to the target functions replaced by the BMI.

## What is the function of the ongoing activity of the brain?

Invasive BMIs will be continuously introduced to use in daily life and should function to voluntarily control moving and resting external devices. Therefore, a principal mystery that BMI research requires present neuroscience to solve is, as Velliste et al. (2014) has discussed, how the brain is active during lengthy periods of behavioral inactivity when no specific tasks are, at least consciously, being performed. This is the problem of the “ongoing” or “intrinsic” activity of the brain (Vincent et al., 2007). Most neuroscience studies have not paid any attention to this problem and have devoted themselves to recording and analyzing neural activity only during the performance of various behavioral tasks. In such recording studies using behavioral tasks, most researchers have implicitly assumed that the spontaneous neural activity prior to the presentation of stimuli or motor responses is an independent random process and have treated it as “baseline activity” or “background noise” unrelated to information processing.

However, this view of spontaneous activity was challenged as early as the 1990s. For example, Arieli et al. (1995) reported that collective ensembles of activity of many neurons of the visual cortex occurred not only during stimulus-evoked periods but also during spontaneous non-stimulus periods. They suggested that the population activity of neurons is not an independent random process even during baseline periods. The correlated activity was detected among neurons comprising a population and among separate populations of neurons. It can be considered that these temporally correlated neurons and populations are cell assemblies, and the ongoing spontaneous activity of cell assemblies may reflect the processing of the context, which affects the processing of incoming sensory stimuli or motor responses (Arieli et al., 1995). The result of Sakurai (1996a) supports this notion, because the correlated activity of hippocampal and auditory cortical neurons, recorded during non-stimulus intertrial intervals, represented the context, i.e., the type of tasks that the animal was currently engaged in. These studies strongly indicate that important mechanisms underlying the higher integrative processing of perception, cognition, attention, and memory depend on the spatiotemporal interactions between ongoing and event-evoked activities of neurons and cell assemblies.

The significant role of the spontaneous activity of neuronal populations may be related to the assumption of “default mode network” suggested by noninvasive imaging (PET, fMRI) studies on human (Raichle, 2010) and monkey (Mantini et al., 2011) brains. The noninvasive images, which represent the activity of large populations of neurons, often show periodic synchronous activation across several close and distant cortical areas during periods of rest with no tasks. Raichle (2010, 2011) suggests that such periodic activation in multiple areas, the “default mode”, has a preparatory function to process incoming sensory stimuli or motor responses. Therefore, the default mode and the ongoing activity have the same features and functions and indicate the significant role of the synchronous activity during periods with no tasks. Further clarification of its functional role must contribute to further development of BMIs that can be mounted continuously in daily life.

## How does the bodily behavior affect the brain function?

The brain controls behavioral functions of the body and, at the same time, the behavior of the body affects the activity of the brain (Chiel and Beer, 1997). In this notion of brain-body interaction, the problem of how bodily movements constrain brain activity is closely related to BMIs, because BMIs require the replacement of bodily movements with machine devices. Typical evidence of the bodily effect on brain activity and function is the “phantom limb” (Ramachandran and Hirstein, 1998). A sudden loss of parts of the body often causes drastic changes in tactile and movement-related perception and generates hallucinations of body images. This confused representation of perceptual information is considered to be due to the reorganization of neuronal networks and the following confused coding of sensory information (Melzack, 1990). Accordingly, a BMI might change functions of the brain, especially the neuronal coding of perceptual information.

The phenomenon of the phantom limb indicates that stable and precise coding in the brain requires stable and precise inputs of sensory information generated from the body. This is not restricted to tactile and movement-related sensations but also applicable to other sensory inputs. “Charles Bonnet syndrome” (Menon et al., 2003) is a typical case of visual hallucinations, i.e., the confused and spontaneous coding of perceptual information caused by the complete and long-lasting deprivation of visual inputs. In addition, isolation experiments cutting off visual, auditory, and tactile sensory inputs, originally discussed by Hebb (1949), showed that several types of hallucination can be generated even in short periods of sensory deprivation (Heron, 1957). If stable and precise sensory inputs are essential for the normal coding of information, the artificial operation of sensory inputs may correct the abnormal hallucinations caused by sensory deprivation. This assumption is supported by the “virtual reality box” experiment (Ramachandran and Hirstein, 1998), in which the artificial presentation of mirror images of lost parts of the body often changes or erases hallucinations that involve body images.

All these findings indicating the importance of sensory inputs consistent with bodily behaviors recommend the further development of BMIs equipped with sensory feedback contingent with the behaviors of brain-controlling devices. Recently, Tabot et al. (2013) have succeeded in restoring tactile feedback using a brain-controlled prosthetic hand. O’Doherty et al. (2011) have developed a BMBI (brain-machine-brain interface), which can provide a monkey with not only visual but also tactile feedback from a brain-controlled device (virtual hand). Further neuroscience research on brain-body interaction will contribute to development of BMBIs, and progress in BMBI research will clarify how the brain interacts with the body, encodes sensory information, and constructs body images (Shokur et al., 2013).

## To what extent can the brain induce plasticity?

This final section further discusses the BMI-induced plasticity of the brain and emphasizes why it is inevitable in all BMIs. Some studies have reported clear changes in the plasticity of neuronal activities and functions induced by the use of BMIs (e.g., Zacksenhouse et al., 2007; Ganguly et al., 2011). Such plastic changes can be thought to be induced to some extent in most BMI experiments, in which the conversion of neuronal signals is aided by appropriate transform algorithms to generate suitable control parameters. The conversion parameters obtained for one set of trials provided increasingly poor predictions of future responses, indicating the drift of neuronal signals over tens of minutes. Therefore, accurate device control under BMI conditions inevitably requires the neuronal activity to be volitionally modulated to become more suitable signals for device control, and the brain surely responds to the request for activity modulation. The BMI-induced changes in neuronal activity are not restricted to the regions from which signals used for device control are recorded. Koralek et al. (2012, 2013) investigated the role of corticostriatal plasticity, usually involved in learning physical skills, in abstract skill learning by a BMI using motor cortical neurons. During the learning of control by the BMI, an alteration of activity was observed in the striatal neurons, and strong correlations, reflected in oscillatory coupling, between the neuronal activity in the motor cortex and the striatum emerged. The authors concluded that temporally precise coherence develops specifically in motor output-related neuronal populations during learning and that the oscillatory activity serves to synchronize widespread brain networks to produce appropriate behaviors.

Discussion of the mechanisms of BMI-induced plastic changes in neuronal activity also requires a psychological view, i.e., operant conditioning. In most BMI situations, the successful control of devices can function as a reward and reinforces the occurrence of volitionally modulated neuronal activity to control the devices. This process of reinforcing the volitional modulation of activity is the operant conditioning of neuronal activity (Fetz, 1969). All BMIs are thought to include this process of conditioning (Fetz, 2007; Sakurai et al., 2014), making plastic changes in neuronal activity inevitable. This leads to the argument that the investigation of neuronal operant conditioning (neurofeedback) will inevitably contribute to the realization of higher-performing BMIs (Fetz, 2007; Moritz et al., 2008; Sakurai et al., 2014). In addition, research on the operant conditioning of synchrony and oscillation of neurons (Engelhard et al., 2013; Fetz, 2013; Sakurai and Takahashi, 2013), i.e., the activity of cell assemblies, will also significantly contribute to the development of BMIs (Sakurai et al., 2014).

It should be noted that the neuronal plasticity inherent in BMI experiments is not always an obstacle for the development of higher-performing BMIs and can be actively applied to research on the extent to which the brain can change and how the brain can be changed efficiently. The former means that BMI studies are able to classify the real plasticity of the brain. The latter suggests that the development of BMIs will lead to the development of better methods of neurorehabilitation to induce changes in neuronal activities and connections for functional compensation (Dobkin, 2007; Fetz, 2007; Jackson and Fetz, 2011; Miller and Weber, 2011).

## Conclusion

**Figure 1:**
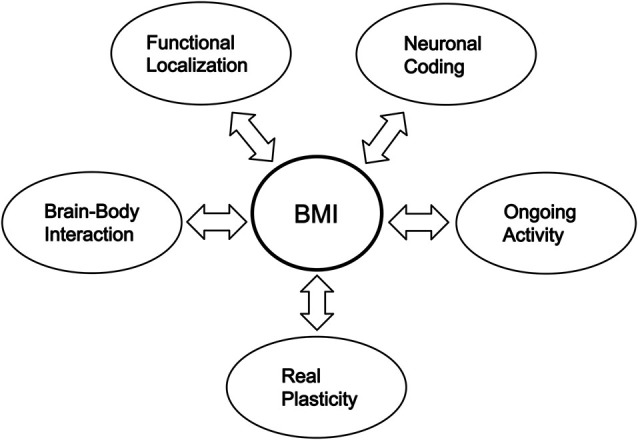
**Interactive advancement of researches on BMIs and the principal mysteries of the brain**.

Although research in modern neuroscience has made great progress, BMI research has shown that we still do not fully understand even the major properties of the brain, i.e., the principal mysteries enumerated in the present paper. BMI research has the role of impelling present neuroscience to clarify the major properties, including the real plasticity, of the brain, and further progress in neuroscience in uncovering the properties of the brain contributes to the further development of BMIs (Figure 1).

## Conflict of interest statement

The author declares that the research was conducted in the absence of any commercial or financial relationships that could be construed as a potential conflict of interest.
